# Effects of Genetic Variants in *ADCY5*, *GIPR*, *GCKR* and *VPS13C* on Early Impairment of Glucose and Insulin Metabolism in Children

**DOI:** 10.1371/journal.pone.0022101

**Published:** 2011-07-15

**Authors:** Jan Windholz, Peter Kovacs, Anke Tönjes, Kathrin Dittrich, Susann Blüher, Wieland Kiess, Michael Stumvoll, Antje Körner

**Affiliations:** 1 University Hospital for Children and Adolescents, University of Leipzig, Leipzig, Germany; 2 Interdisciplinary Center for Clinical Research, University of Leipzig, Leipzig, Germany; 3 Department of Medicine, University of Leipzig, Leipzig, Germany; 4 Leipzig University Medical Center, IFB Adiposity Diseases, University of Leipzig, Leipzig, Germany; German Diabetes Center, Leibniz Center for Diabetes Research at Heinrich Heine University, Germany

## Abstract

**Objective:**

Recent genome-wide association studies identified novel candidate genes for fasting and 2 h blood glucose and insulin levels in adults. We investigated the role of four of these loci (*ADCY5*, *GIPR*, *GCKR* and *VPS13C*) in early impairment of glucose and insulin metabolism in children.

**Research Design and Methods:**

We genotyped four variants (rs2877716; rs1260326; rs10423928; rs17271305) in 638 Caucasian children with detailed metabolic testing including an oGTT and assessed associations with measures of glucose and insulin metabolism (including fasting blood glucose, insulin levels and insulin sensitivity/secretion indices) by linear regression analyses adjusted for age, sex, BMI-SDS and pubertal stage.

**Results:**

The major allele (C) of rs2877716 (*ADCY5*) was nominally associated with decreased fasting plasma insulin (P = 0.008), peak insulin (P = 0.009) and increased QUICKI (P = 0.016) and Matsuda insulin sensitivity index (P = 0.013). rs17271305 (*VPS13C*) was nominally associated with 2 h blood glucose (P = 0.009), but not with any of the insulin or insulin sensitivity parameters. We found no association of the *GIPR* and *GCKR* variants with parameters of glucose and insulin metabolism. None of the variants correlated with anthropometric traits such as height, WHR or BMI-SDS, which excluded potential underlying associations with obesity.

**Conclusions:**

Our data on obese children indicate effects of genetic variation within *ADCY5* in early impairment of insulin metabolism and *VPS13C* in early impairment of blood glucose homeostasis.

## Introduction

Although obesity is the most important risk factor for disorders of glucose metabolism such as type 2 diabetes (T2D), the risk for those metabolic disorders is modified by an individual's genetic predisposition. Recent genome wide association studies (GWAS) have identified several genetic loci associated with fasting blood glucose (FBG), 2 h blood glucose (BG) and insulin secretion in adults [1], [2]. In a meta analysis of GWAS on 2 h BG after an oral glucose challenge, three novel loci (*GIPR*, *ADCY5* and *VPS13C*) have emerged to be of highest significance and potential importance in adults (2). Of the previously known genes associated with type 2 diabetes (T2D), variants in *GCKR* and *TCF7L2* were confirmed to be associated with 2 h BG [2]. However, most of these and subsequent replication studies were performed in adults. Since children are much less biased by comorbidities and their medication, and considering that the exposure to environmental factors, particularly obesity, is much shorter, they are an attractive population for identifying primary genetic determinants controlling complex polygenic traits.

We previously showed that diabetes susceptibility variants in *TCF7L2* were associated with early impairment of glucose tolerance in children before they develop overt diabetes [3]. Here, we aimed to elucidate the role of four new recently identified loci (*GCKR*, *GIPR*, *ADCY5* and *VPS13C*) in early impairment of glucose and insulin metabolism by investigating their effects in a cohort of obese Caucasian children.

## Methods

### Subjects

#### Ethics Statement

Written informed consent was obtained from all guardians and children older 12 years. The study was approved by the ethical committee of the University of Leipzig.

#### Cohort design

We recruited 571 Caucasian children and adolescents from our obesity clinic (Leipzig, Germany) with a BMI≥1.23 SDS (standard deviation score). This cohort included 61 overweight (1.88 SDS≥BMI≥1.23 SDS) subjects. In addition, we included 67 healthy lean controls from our Leipzig Atherobesity Childhood cohort [4]. We excluded participants with antidiabetic medications (metformin) prior to analysis. Height was measured to the nearest of 0.1 cm and weight to the nearest of 0.1 kg using a digital scale. Body mass index (BMI) data were standardized to age and sex of the children applying German reference data [5], and is given as BMI standard deviation score (SDS). Demographic characteristics of the study cohort are given in Table 1. Blood lipids, glucose and insulin were measured by a certified laboratory applying standard clinical biochemistry methods.

**Table 1:** Demographic characteristics of study population.

Variable	Mean±SD
n (girls/boys)	638 (333/305)
Age (years)	11.5±3.6
BMI (kg/m^2^)	28.0±6.7
BMI SDS	2.26±1.10
WHR	0.87±0.08
PH	2.65±1.74

We performed a comprehensive metabolic work-up, including lipid profiles, an oral glucose tolerance test (oGTT) and blood pressure measurements in all children. For oGTT, blood samples were collected at 0, 30, 60, 90 and 120 minutes after the glucose load of 1.75 g per kg body weight (maximum of 75 g glucose). We calculated several insulin sensitivity and resistance indices to assess glucose homeostasis in children (precise equations are given in Method S1). The prevalence of impaired FBG was 9.0% and the prevalence of impaired glucose tolerance was 15.3% in our sample.

#### Genotyping

Four variants (rs2877716, rs1260326, rs10423928, rs17271305) were genotyped using TaqMan allelic discrimination assays (Applied Biosystems, Inc., Foster City, CA) on ABI PRISM 7500 sequence detector (Applied Biosystems). Genotyping reproducibility was assessed with a random 5% selection of the samples, which were re-genotyped for all variants and we obtained 100% concordance.

#### Statistical analyses

We performed logarithmic transformation with logarithmus naturalis (ln) for measures that were not normally distributed. To test for associations of genetic variants with quantitative traits of metabolic and anthropometric characteristics we applied generalized linear regression models under the additive mode of inheritance adjusted for age, sex, pubertal stage and BMI SDS. Pubertal stage is clinically assessed by Tanner stage ranging from PH1 ( = prepubertal) to PH5 ( = adolescent, puberty completed). Hence, we regarded the PH as an ordinal variable.

A *P*-value<0.05 was considered to provide *nominal* evidence for association. Considering four SNPs and two traits (glucose, insulin) being analysed, a *P*-value<0.00625 would indicate significance after Bonferroni corrections for multiple testing. Power calculations for BMI SDS and metabolic traits for all SNPs are given in Supplementary Table S1.

Statistical analyses were performed using the SPSS software package (version 18.0) (SPSS, Inc., Chicago, IL).

## Results

We evaluated associations of the four variants (minor allele frequency) rs2877716 (0.21), rs1260326 (0.40), rs10423928 (0.22), rs17271305 (0.37) in 638 children. Genotyped allelic frequencies were consistent with Hardy-Weinberg equilibrium for all variants. Data on metabolic traits stratified by genotypes are given in Table 2.

**Table 2:** Metabolic characteristics stratified for the gene variants.

	*rs2877716 (ADCY5)*			*rs1260326 (GCKR)*			*rs10423928 (GIPR)*			*rs17271305 (VPS13C)*		
	CC (61.7%)	CT (33.3%)	TT (5%)	CC (36.8%)	CT (47.3%)	TT (14.9%)	TT (60%)	AT (34.1%)	AA (5.2%)	AA (40.8%)	AG (43.5%)	GG (13.6%)
BMI SDS	2.23±0.05	2.34±0.07	2.28±0.21	2.26±0.07	2.22±0.06	2.34±0.11	2.22±0.06	2.3±0.07	2.38±0.16	2.28±0.07	2.25±0.06	2.33±0.1
FBG	4.6±0.02	4.6±0.03	4.7±0.1	4.6±0.03	4.6±0.03	4.6±0.05	4.6±0.03	4.6±0.03	4.6±0.1	4.6±0.03	4.6±0.03	4.6±0.05
BG (2 h)	5.8±0.05	5.8±0.07	5.5±0.2	5.8±0.06	5.8±0.06	5.7±0.1	5.7±0.05	5.9±0.06	5.5±0.2	5.7±0.06	5.8±0.06	6±0.1
BG AUC	733±7	739±9	698±27	729±9	733±7	736±11	734±7	736±8	695±28	728±8	732±8	751±13
FPI^a^	77±2	84±3	96±12	80±3	79±3	82±5	80±3	79±3	93±10	81±3	80±3	79±5
Insulin Peak^a^	933±34	1018±50	1075±111	974±43	936±38	1018±81	982±36	955±46	917±105	988±49	955±38	971±67
Insulin AUC^a^	68743±2663	73793±3563	74484±7987	70497±3345	67924±2795	77322±6452	71818±2605	69619±3717	66699±8492	72470±3700	69275±2805	70630±5342
HOMA-Ir^a^	2.2±0.07	2.4±0.1	2.8±0.4	2.3±0.1	2.3±0.08	2.4±0.2	2.3±0.08	2.3±0.1	2.7±0.3	2.4±0.1	2.3±0.09	2.3±0.2
HOMA-B^a^	320±125	244±14	314±55	210±12	207±16	273±38	215±14	464±227	159±80	429±202	203±15	238±23
QUICKI^a^	0.4±0.002	0.3±0.002	0.3±0.006	0.4±0.002	0.4±0.002	0.3±0.004	0.4±0.002	0.4±0.003	0.3±0.005	0.4±0.002	0.4±0.002	0.3±0.004
ISI^a^	6.1±0.2	5.2±0.2	4.8±0.5	5.7±0.3	5.9±0.2	5.5±0.4	5.7±0.2	6±0.3	4.7±0.4	5.8±0.2	5.8±0.2	5.5±0.4
Belfiore^a^	0.6±0.02	0.6±0.02	0.5±0.05	0.6±0.02	0.6±0.02	0.6±0.03	0.6±0.02	0.6±0.02	0.6±0.05	0.6±0.02	0.6±0.02	0.6±0.04
Gutt^a^	58±1	72±15	68±10	70±13	59±1	63±4	59±1	73±14	57±3	58±1	69±10	62±5
Stumvoll	−0.3±0.004	−0.3±0.007	−0.3±0.01	−0.3±0.006	−0.3±0.005	−0.3±0.009	−0.3±0.005	−0.3±0.006	−0.3±0.01	−0.3±0.005	−0.3±0.006	−0.3±0.01
IGI^a^	49±7	47±5	61±8	50±4	49±9	47±3	54±7	40±6	50±6	46±2	40±4	86±31
Ratio AUC_BG_/AUC_Insulin_	95±4	98±4	102±10	97±5	93±4	101±7	98±4	94±5	92±10	98±5	96±4	92±6

Data are given for each individual genotype as means ± SEM. FBG: Fasting blood glucose (mmol/l); BG: blood glucose; FPI: fasting plasma insulin (pmol/L); AUC: Area under the curve; for a detailed description of the Insulin resistance and –sensitivity indices see Method S1.

### Association of genotypes with parameters of obesity

We did not observe a significant association of any of the variants with BMI SDS or other surrogate markers of obesity such as skinfold or WHR (all *P*>0.2) (Table 3). Also, there was no association with birth weight. Results were concordant irrespective of the genetic model (additive/dominant) applied.

**Table 3:** Effect sizes and P values for metabolic parameters according to genotype.

	*rs2877716 (ADCY5)*				*rs1260326 (GCKR)*				*rs10423928 (GIPR)*				*rs17271305 (VPS13C)*			
	Beta^1^	P^1^	Beta^2^	P^2^	Beta^1^	P^1^	Beta^2^	P^2^	Beta^1^	P^1^	Beta^2^	P^2^	Beta^1^	P^1^	Beta^2^	P^2^
BMI SDS	−0.1	0.162			−0.02	0.701			−0.08	0.238			−0.03	0.681		
FBG	<0.01	0.932	<0.01	0.963	0.02	0.443	0.02	0.426	0.03	0.369	0.03	0.314	<0.01	0.944	<0.01	0.928
BG (2 h)	0.06	0.400	0.08	0.245	0.03	0.662	0.03	0.588	−0.01	0.897	0.01	0.903	−0.16	0.009	−0.15	0.009
BG AUC	5.16	0.554	7	0.419	−4.41	0.562	−4.27	0.570	6.9	0.428	8.42	0.329	−8.35	0.269	−8.26	0.270
FPI^a^	−0.1	0.008	−0.08	0.024	−0.01	0.664	−0.01	0.769	−0.05	0.246	−0.03	0.384	0.01	0.667	0.02	0.527
Insulin Peak^a^	−0.11	0.009	−0.09	0.023	<0.01	0.980	0.01	0.884	0.02	0.710	0.03	0.466	0.01	0.770	0.01	0.691
Insulin AUC^a^	−0.09	0.034	−0.07	0.075	−0.01	0.709	−0.01	0.742	0.03	0.471	0.05	0.255	0.02	0.551	0.03	0.452
HOMA-Ir^a^	−0.1	0.015	−0.08	0.043	−0.01	0.775	−0.01	0.864	−0.04	0.352	−0.02	0.510	0.02	0.653	0.02	0.538
HOMA-B^a^	−0.14	0.004	−0.12	0.010	−0.04	0.324	−0.03	0.355	−0.08	0.098	−0.07	0.126	0.02	0.552	0.03	0.473
QUICKI^a^	0.02	0.016	0.01	0.045	<0.01	0.766	<0.01	0.854	0.01	0.342	<0.01	0.497	<0.01	0.693	<0.01	0.575
ISI^a^	0.1	0.013	0.08	0.035	0.02	0.633	0.01	0.701	0.02	0.651	0.01	0.871	−0.01	0.860	−0.01	0.757
Belfiore^a^	0.07	0.081	0.06	0.164	0.04	0.320	0.03	0.357	−0.03	0.540	−0.04	0.333	−0.01	0.718	−0.02	0.629
Gutt^a^	−0.01	0.641	−0.02	0.386	−0.01	0.548	−0.01	0.463	−0.01	0.619	−0.02	0.434	−0.01	0.641	−0.01	0.545
Stumvoll	<0.01	0.746	<0.01	0.459	<0.01	0.931	<0.01	0.927	<0.01	0.651	<0.01	0.982	0.01	0.324	0.01	0.355
IGI^a^	−0.1	0.050	−0.08	0.093	<0.01	0.956	<0.01	0.958	0.03	0.484	0.05	0.334	−0.03	0.535	−0.03	0.542
Ratio AUC_BG_/AUC_Insulin_ a	−0.08	0.043	−0.07	0.086	−0.01	0.820	−0.01	0.815	0.03	0.456	0.04	0.280	0.03	0.390	0.03	0.320

All data is adjusted to age, sex and pubertal state. Beta is non-standardized per-allele beta (SE) in the additive model and refers to the major allele. FBG: Fasting blood glucose (mmol/l); BG: blood glucose; FPI: fasting plasma insulin (pmol/L); AUC: Area under the curve; For a detailed description of the Insulin resistance and –sensitivity indices see Method S1.

1 adjusted for age, sex and pubertal stage;

2 adjusted for age, sex, pubertal stage and BMI SDS;

a for non-normally distributed variables, data were log-transformed.

### Association of genotypes with insulin and glucose traits

**Figure 1:**
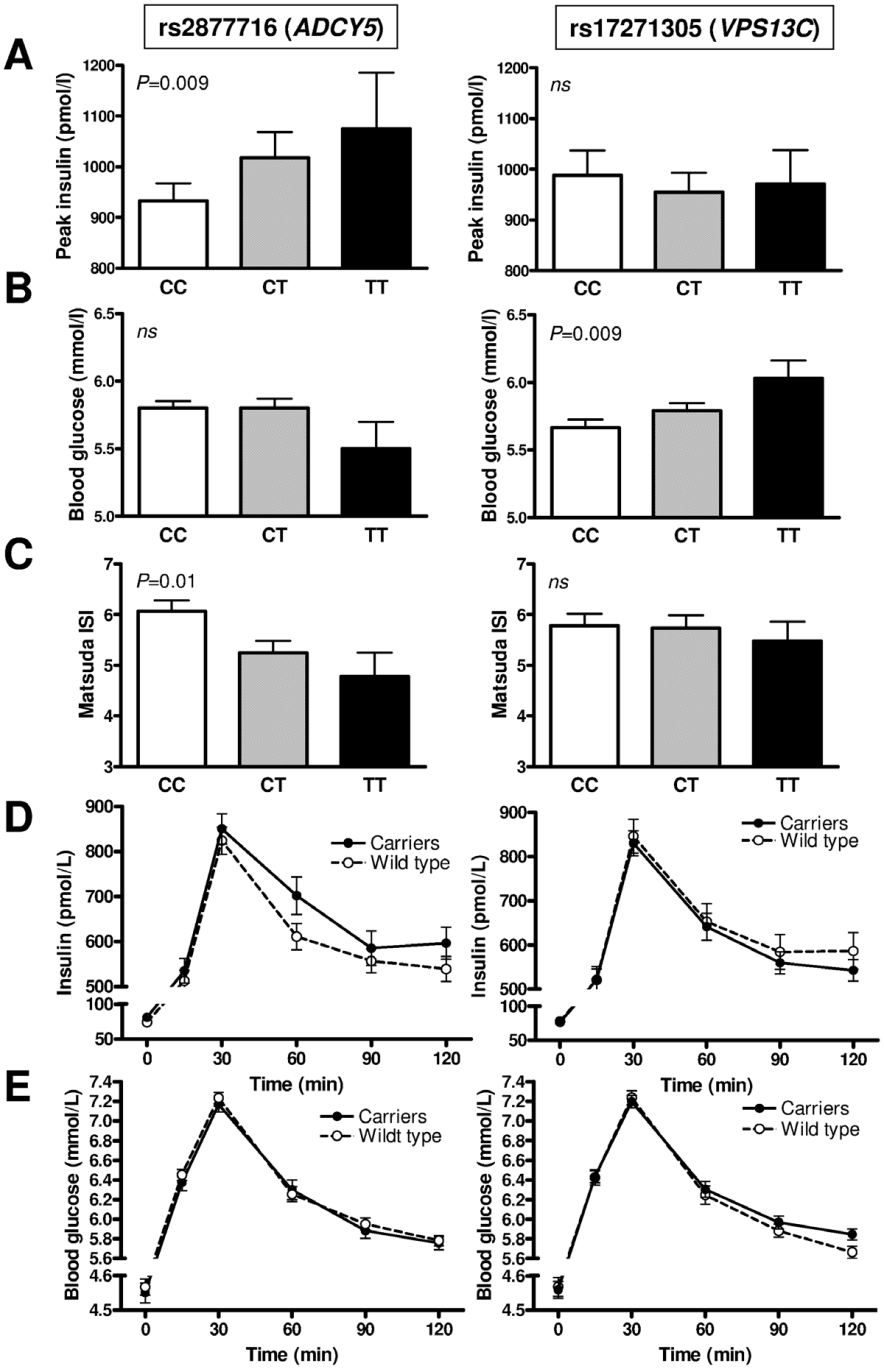
Association of rs2877716 (*ADCY5*) and rs17271305 (*VPS13C*) risk alleles with metabolic parameters. Peak insulin (**A**), 2 h blood glucose (**B**) and Matsuda insulin sensitivity index (**C**) are given for respective genotypes of rs2877716 (left) and rs17271305 (right). Dynamic course of insulin (**D**) and blood glucose (**E**) are given for carriers of the risk allele and wild types as indicated. The effects determined by oral glucose tolerance test were evaluated using generalized linear regression models applying the additive model of inheritance as indicated. The analysis was adjusted for age, sex and pubertal stage. Data are shown as mean±SEM.

The C-allele in rs2877716 (*ADCY5*) was nominally associated with parameters of insulin secretion and insulin resistance, such as a lower fasting plasma insulin, peak insulin (Fig. 1A), HOMA-B and a higher QUICKI and ISI (Fig. 1C), but showed no impact on blood glucose parameters (Fig. 1B) (Table 1). The associations were preserved after additional adjustment for BMI-SDS and hence independent of obesity (Table 3).

The A-allele in rs17271305 (*VPS13C*) was significantly associated with a lower 2 h BG (Fig. 1B). Again, after adjustment for BMI SDS, the association became weaker but remained nominal (Table 3).

Plotting the genotype stratified response to oral glucose illustrated the association of rs2877716 (*ADCY5*) with insulin response, while for rs17271305 (*VPS13C*) the glucose response was different according to genotype (Fig. 1D+E).

The variants in *GIPR* and *GCKR* did not show any significant association with insulin and glucose or with insulin resistance/sensitivity indices (Table 3).

We did not find any significant relationships between any of the variants and cholesterol levels, liver enzymes or serum leptin concentrations.

## Discussion

In this study we show a nominal association of rs2877716 (*ADCY5*) with insulin secretion and of rs17271305 (*VPS13C*) with 2 h BG in children. Other variants previously associated with glucose homeostasis in adults were, however, not associated with parameters of glucose or insulin metabolism in children.

To our knowledge these results are the first data shown for children. Our finding in a cohort with a mean age of 11.5 years and hence shorter exposure to obesity and other adverse (life style) factors, supports a role of *ADCY5* in the pathophysiology of insulin resistance, independent of obesity. The biological function of adenylate cyclase 5 is diverse. It is a member of the adenylate cyclase family, which consists of twelve domain transmembrane proteins that catalyzes the conversion of ATP to cyclic AMP, the second messenger for G protein coupled receptors. The ADCY isoform 5 has been described to mediate renin release [6], influence heart rate [7] and interact with dopamine homeostasis in the central nervous system [8], hence particularly affecting endocrine/metabolic and cardiovascular signaling. Adjusting analyses for BMI SDS somewhat diminished the magnitude of the effect; although it was still significant. Considering the strong impact of obesity on insulin metabolism, an additional contribution of BMI to hyperinsulinemia may be possible. It is well known that children go through a phase of (physiological) insulin resistance during mid-puberty [9]. We have, therefore, adjusted our analyses for pubertal stage in addition to age and the observed associations are hence unbiased by this phenomenon.

Interestingly, variants in *ADCY5* were also found to be associated with birth weight and fetal growth [10]. Since lower birth weight itself is a risk factor for type 2 diabetes [11], an interaction of rs2877716 with birth weight and glucose homeostasis could be hypothesized. We did, however, not observe any relationship between rs2877716 genotype and birth weight or with current BMI or other obesity measures in our cohort. Therefore, the association of rs2877716 with insulin secretion does not appear to be secondary to an underlying association of rs2877716 with birth weight or with obesity. Also, the analysis of the association of rs2877716 with insulin secretion parameters in the lean subcohort did reveal significant association, although we are aware of the limited sample size not allowing conclusive evidence from this subcohort.

Our data show that presence of the C-allele in rs2877716 results in lower glucose stimulated plasma insulin and consequently on insulin derived indices. This does, however, not lead to higher blood glucose implicating that glucose homeostasis in these children still being preserved. We can, however, not explain the U-shaped distribution of phenotypes for the HOMA-B. However, the other parameters (e.g. HOMA-IR, insulin levels) show increasing values with the number of minor (T-) alleles indicating that the C-allele is associated with decreased peak insulin and better insulin sensitivity. In previous studies in adults the effects of rs2877716 was consistent with our data. Dupuis et al. showed the same effect for rs11708067, which is in 95% Linkage disequilibrium with rs2877716, and described a nominal association of the major allele with a lower fasting insulin and with higher 2 h BG [1].

The sequence of events of obesity with subsequent insulin resistance and consequently hyperinsulinemia is understood in that increased post-challenge glucose levels result from the deviation of the hyperbolic relationship between insulin sensitivity and insulin plasma levels and is hence a sign of decompensation of β-cell function [12], [13]. While there is a gradual but continuous decline in β-cell function, glucose levels rise relatively late in this pathologic process. Hence, considering that children represent an earlier stage of pathology, it is not entirely unexpected, that we do see a change in insulin levels, while glucose levels are still relatively preserved. Hence, the association of the *ADCY5* SNP may still be related to the complex glucose-insulin-pathophysiology, but not exclusively to glucose levels (as concluded from adult studies).

Ingelsson et al. performed a detailed characterization with discrimination of insulin processing, secretion and sensitivity [14]. Their results indicate that *GIPR* is associated with a lower insulin secretion (represented by insulinogenic index), *GCKR* with a reduced insulin sensitivity (represented by Stumvoll, Matsuda and Belfiore indices), while *VPS13C* was only modestly associated with higher proinsulin levels and *ADCY5* did not show any obvious effect on insulin sensitivity or secretion in adults.

A common assumption is that children, unlike adults, are less influenced by prolonged exposure to environmental factors and obesity related comorbidities and therefore allow discriminating primary genetic determinants assumed to effect complex polygenic diseases like obesity. Nevertheless, the variant-effect may manifest in later stages of life, potentially additionally modified by gene-environment interactions [15], or may be detectable only with advanced stages of disease, which are less prevalent in children. In consequence, obese children present with earlier stages of metabolic derangement and the increase in insulin levels precedes the deterioration of blood glucose allostasis [16]. Nevertheless, considering our observed associations with insulin parameters and that insulin parameters are the first to be affected in obese children, one may conclude that rs2877716 is affecting insulin secretion and/or resistance at an early stage.

The variant in the *VPS13C* gene showed a nominal association with blood glucose, but unexpectedly no association with insulin secretion or indices of insulin resistance. The family of *VPS13C* plays a role in transport of membrane proteins between trans-Golgi network and the prevacuolar compartment [17] and is highly expressed in beta cells [2]. However, the precise function of *VPS13C* is unknown. Saxena et al. found rs17271305 to be associated with 2 h BG and lower 2 h insulin, but not with T2D. We were able to replicate the association with 2 h BG already in children. Also similar to adults, the insulin response to oral glucose was not different between carriers of the risk allele and wild types.

Finally, we did not detect any effect of variants in *GIPR* or *GCKR* with glucose metabolism in this pediatric study. Again, we may speculate that this lack of significance is due to the less prevalent overt phenotype in children, or that the effect of these variants is not as strong as other genes.

We are aware that our study has several limitations. Even though we did achieve nominal associations for the SNPs in *ADCY5* and *VPS13C*, when each SNP and trait was considered separately, the association would not withstand correction for Bonferroni multiple testing with a P value of 0.00625 as a threshold of statistical significance. This is most likely due to the small sample size. Given the observed allele frequencies for the respective SNPs, we achieved 80% statistical power to detect effect sizes ranging from 0.077 to 0.09 for FBG and from 0.16 to 0.19 mmol/L for 2 h BG in the additive model. We may, therefore, have missed associations for smaller effects. In the paper by Saxena et al. [2], the observed effect size was 0.09 mmol/L per allele for 2 h BG for *ADCY5*, which is indeed beyond the limit that we were able to detect in our cohort, and which may partially explain why we see nominal positive associations for only few insulin and glucose determinants. Nevertheless, considering the observed associations with insulin parameters and that insulin parameters are the first to be affected in obese children, one may conclude that this SNP is affecting insulin secretion and/or resistance at an early stage. It is also noteworthy that consistent with the discovery GWAS [1], [2] suggesting additive mode of inheritance for the allele affects, we applied the same model in our analyses despite the small number of homozygous subjects for the minor alleles e.g. at *ADCY5* and *GIPR*.

In summary, we provide evidence that in children genetic variations within *ADCY5* and *VPS13C* contribute to early impairment of insulin metabolism and early impairment of blood glucose homeostasis, respectively.

## Supporting Information

Method S1 Calculation of Insulinresistance-Indices.
(DOC)

Table S1 **Power calculations.** SNP effect sizes detectable with 80% at α = 0.05.
(DOC)
