# Supplementary material for: Effects of Genetic Variants in ADCY5, GIPR, GCKR and VPS13C on Early Impairment of Glucose and Insulin Metabolism in Children
Source: PLoS One. 2011 Jul 15;6(7):e22101. doi: 10.1371/journal.pone.0022101 (PMC3137620; doi:10.1371/journal.pone.0022101)
Supplement: Method S1 — Calculation of Insulinresistance-Indices. (DOC) [file pone.0022101.s001.doc]

## Supplementary Methods: Calculation of Insulinresistance-Indices

HOMA-B (Homeostasis Model Assessment – Beta-Cell Function) (1)

(fasting plasma insulin [µU / ml] * 20) / (fasting blood glucose [mmol/l] – 3.5)

HOMA-IR (Homeostasis Model Assessment) (1)

(fasting blood glucose [mmol/l] * fasting plasma insulin [µU / ml]) / 22.5

QUICKI (Quantitative Insulin Sensitivity Check Index) (2)

1 / (log fasting blood glucose [mmol/l] + log fasting plasma insulin [µU / ml])

ISI (Insulinsensitivity-Index) (3)

10000/SQRT(* fasting plasma insulin [µU / ml] * mean blood glucose [mmol/l] * mean plasma insulin [µU / ml])

Belfiore-Index (4)

2/((((0.5* fasting blood glucose [mmol/l]) + 60 min mean blood glucose [mmol/l] +(0.5*120 min blood glucose [mmol/l]) *(((0.5* fasting plasma insulin [µU / ml]) + 60min plasma insulin [µU / ml] + (0.5* 120 min plasma insulin [µU / ml])) / 638) + 1)

Gutt-Index (5)

=(75000 + (fasting blood glucose [mmol/l] – 120 min blood glucose [mmol/l] * 0.19 * weight [kg] / 120) / ((fasting blood glucose [mmol/l] + 120 min blood glucose [mmol/l] / 2) / LOG_10((fasting plasma insulin [µU / ml] + 120 min plasma insulin [µU / ml]) / 2)

Stumvoll-Index (6)

0.226-(0.0032 * BMI [kg/m²])-(0.0000645 * 120 min plasma insulin [µU / ml]) - (0.00375 * 90 min blood glucose [mmol/l])

Area-under-the-curve (AUC) (7)

We computed the Area under the curve using trapezoidal integration at 0, 30, 60, 90, 120 min.

Area-under-the-curve quotient (7)

AUC fasting plasma insulin [pmol/l] /AUC fasting blood glucose [mmol/l]

Insulinogenic index (IGI) (3)

= (30 min plasma insulin – fasting plasma insulin) [µU/ml] / (30 min blood glucose – fasting blood glucose) [mmol/l]

## References for supplementary methods

1. **Matthews DR, Hosker JP, Rudenski AS, Naylor BA, Treacher DF, Turner RC**. Homeostasis model assessment: insulin resistance and beta-cell function from fasting plasma glucose and insulin concentrations in man. Diabetologia 1985; 28(7):412-419.

2. **Katz A, Nambi SS, Mather K, Baron AD, Follmann DA, Sullivan G, Quon MJ**. Quantitative insulin sensitivity check index: a simple, accurate method for assessing insulin sensitivity in humans. J Clin Endocrinol Metab 2000; 85(7):2402-2410.

3. **Matsuda M, DeFronzo RA**. Insulin sensitivity indices obtained from oral glucose tolerance testing: comparison with the euglycemic insulin clamp. Diabetes Care 1999; 22(9):1462-1470.

4. **Belfiore F, Iannello S, Volpicelli G**. Insulin sensitivity indices calculated from basal and OGTT-induced insulin, glucose, and FFA levels. Mol Genet Metab 1998; 63(2):134-141.

5. **Gutt M, Davis CL, Spitzer SB, Llabre MM, Kumar M, Czarnecki EM, Schneiderman N, Skyler JS, Marks JB**. Validation of the insulin sensitivity index (ISI(0,120)): comparison with other measures. Diabetes Res Clin Pract 2000; 47(3):177-184.

6. **Stumvoll M, Mitrakou A, Pimenta W, Jenssen T, Yki-Jarvinen H, Van Haeften T, Renn W, Gerich J**. Use of the oral glucose tolerance test to assess insulin release and insulin sensitivity. Diabetes Care 2000; 23(3):295-301.

7. **Matthews JN, Altman DG, Campbell MJ, Royston P**. Analysis of serial measurements in medical research. BMJ 1990; 300(6719):230-235.
