# Supplementary material for: Effects of Genetic Variants in ADCY5, GIPR, GCKR and VPS13C on Early Impairment of Glucose and Insulin Metabolism in Children
Source: PLoS One. 2011 Jul 15;6(7):e22101. doi: 10.1371/journal.pone.0022101 (PMC3137620; doi:10.1371/journal.pone.0022101)
Supplement: Table S1 — Power calculations. SNP effect sizes detectable with 80% at α = 0.05. (DOC) [file pone.0022101.s002.doc]

Supplementary Table S1. **Power calculations**

| **Parameter** | **ADCY5** (rs2877716) | **GCKR** (rs1260326) | **GIPR** (rs10423928) | **VPS13C** (rs17271305) |
| --- | --- | --- | --- | --- |
| BMI SDS | 0.76 | 0.64 | 0.75 | 0.64 |
| FBG (mmol/L) | 0.090 | 0.077 | 0.089 | 0.078 |
| 2h BG (mmol/L) | 0.19 | 0.16 | 0.185 | 0.16 |
| BG AUC (mmol/L)^2^ | 23.5 | 19.5 | 23 | 20 |
| FPI (pmol/L) | 10.75 | 9 | 10.57 | 9.1 |
| Insulin Peak (pmol/L) | 134 | 111 | 132 | 113 |
| Insulin AUC (pmol/L)^2^ | 10000 | 8500 | 10000 | 8500 |
| HOMA-IR | 0.32 | 0.27 | 0.32 | 0.27 |
| QUICKI | 0.0072 | 0.006 | 0.0072 | 0.0061 |
| Matsuda-ISI | 0.75 | 0.62 | 0.74 | 0.63 |
| Ratio AUC_BG_/AUC_Ins_ | 12.5 | 10.5 | 12.5 | 10.5 |
